# Up-to-Date Breast, Cervical, and Colorectal Cancer Screening Test Use in the United States, 2021

**DOI:** 10.5888/pcd20.230071

**Published:** 2023-10-26

**Authors:** Susan A. Sabatino, Trevor D. Thompson, Mary C. White, Maria A. Villarroel, Jean A. Shapiro, Jennifer M. Croswell, Lisa C. Richardson

**Affiliations:** 1 Division of Cancer Prevention and Control, National Center for Chronic Disease Prevention and Health Promotion, Centers for Disease Control and Prevention, Atlanta, Georgia; 2 Division of Health Interview Statistics, National Center for Health Statistics, Centers for Disease Control and Prevention, Hyattsville, Maryland; 3 Division of Cancer Control and Population Sciences, National Cancer Institute, Bethesda, Maryland

## Abstract

**Introduction:**

We examined national estimates of breast, cervical, and colorectal cancer (CRC) screening test use and compared them with Healthy People 2030 national targets. Test use in 2021 was compared with prepandemic estimates.

**Methods:**

In 2022, we used 2021 National Health Interview Survey (NHIS) data to estimate proportions of adults up to date with US Preventive Services Task Force recommendations for breast (women aged 50–74 y), cervical (women aged 21–65 y), and CRC screening (adults aged 50–75 y) across sociodemographic and health care access variables. We compared age-standardized estimates from the 2021 and 2019 NHIS.

**Results:**

Percentages of adults up to date in 2021 were 75.7% (95% CI, 74.4%–76.9%), 75.2% (95% CI, 73.9%–76.4%), and 72.2% (95% CI, 71.2%–73.2%) for breast, cervical, and CRC screening, respectively. Estimates were below 50% among those without a wellness check in 3 years (all screening types), among those without a usual source of care or insurance (aged <65 y) (breast and CRC screening), and among those residing in the US for less than 10 years (CRC screening). Percentages of adults who were up to date with breast and cervical cancer screening and colonoscopy were similar in 2019 and 2021. Fecal occult blood/fecal immunochemical test (FOBT/FIT) use was modestly higher in 2021 (*P* < .001).

**Conclusions:**

In 2021, approximately 1 in 4 adults of screening age were not up to date with breast, cervical, and CRC screening recommendations, and Healthy People 2030 national targets were not met. Disparities existed across several characteristics, particularly those related to health care access. Breast, cervical, and colonoscopy test use within recommended screening intervals approximated prepandemic levels. FOBT/FIT estimates were modestly higher in 2021.

**Summary**

**What is already known on this topic?**

Breast, cervical, and colorectal cancer screening has been below the national 2020 Healthy People targets.

**What is added by this report?**

We used 2021 National Health Interview Survey data to examine the most recent national estimates of screening test use, disparities, and comparisons with 2030 Healthy People targets. Estimates were compared with 2019 estimates to examine differences during the COVID-19 pandemic.

**What are the implications for public health practice?**

Approximately 1 in 4 adults of screening age were not up to date with breast, cervical, and colorectal cancer screening in 2021. Estimates were below current national targets and disparities existed. Test use within recommended intervals may approximate prepandemic levels; fecal occult blood test and fecal immunochemical test use may have increased modestly.

## Introduction

The US Preventive Services Task Force (USPSTF) recommends breast, cervical, and colorectal cancer (CRC) screening to reduce cancer mortality rates (1). Use of these services did not reach national targets for 2020 (2,3). We used 2021 National Health Interview Survey (NHIS) data to examine the most recent national estimates of screening test use, disparities, and comparisons to national targets set by Healthy People (HP) 2030 (4). We compared estimates from 2021 with 2019 estimates to examine differences before and during the COVID-19 pandemic.

## Methods

We used data from the 2021 and 2019 NHIS, an in-person survey of nationally representative samples of the civilian, noninstitutionalized US population (5). One sample adult was randomly selected from each household to self-report information about health and health services use (final sample adult response rates were 50.9% for 2021 and 59.1% for 2019) (5). Because of the COVID-19 pandemic, telephone interviews were attempted first in early 2021. In May 2021, in-person interviews resumed as local conditions allowed (5). Interviews conducted at least partially by telephone were higher in 2021 than in 2019 (62.8% vs 34.3%), and survey weighting methods were modified between these years (5).

We included women aged 50 to 74 years (n = 6,851), women aged 21 to 65 years (n = 10,909), and adults aged 50 to 75 years (n = 12,938) in breast, cervical, and CRC screening analyses, respectively. Exclusions (in order) included prior or unknown hysterectomy (cervical only, n = 1,591), personal or unknown history of the cancer screened for (breast, n = 394; cervical, n = 71; CRC, n = 138), and insufficient information to determine screening status (breast, n = 130; cervical, n = 531; CRC, n = 280). USPSTF updated CRC screening recommendations to include adults aged 45 to 49 years (B recommendation) in May 2021 (6). Because this occurred mid-2021, CRC screening analyses include adults aged 50 to 75 years.

We defined being up to date with screening as reporting having received a USPSTF-recommended test for any reason within recommended screening intervals (Table 1) (1). Each year since the survey redesign in 2019 (www.cdc.gov/nchs/nhis/2019_quest_redesign.htm), NHIS has included a set of cancer control questions sponsored by the Centers for Disease Control and Prevention and the National Cancer Institute. The content of these questions varies from year to year on a rotating basis. In 2019 and 2021, this content included additional questions about breast, cervical, and CRC screening tests to complement rotating core questions in those years about whether respondents ever received these tests and the time since the most recent test. These screening tests included mammography (breast cancer); Papanicolaou (Pap) and human papillomavirus (HPV) tests (cervical cancer); and colonoscopy, sigmoidoscopy, computed tomography (CT) colonography, home fecal occult blood test (FOBT) or fecal immunochemical (FIT) test, and FIT-DNA test (CRC). For blood stool tests, separate questions were not asked about FIT and guaiac tests. Rather, they were asked about in combination (“The following questions are about the blood stool or fecal occult blood test, fecal immunochemical or FIT test. These are tests to determine whether you have blood in your stool or bowel movement and can be done at home using a kit. You use a stick or brush to obtain a small amount of stool at home and send it back to the doctor or lab. Have you ever had a blood stool or FIT test, using a HOME test kit? When was your most recent blood stool or FIT test, using a home test kit?”). In 2019, questions about nonendoscopic CRC screening tests were asked of respondents who answered yes when asked if they had received a CRC test other than colonoscopy or sigmoidoscopy. In 2021, questions about nonendoscopic tests were asked of all respondents aged 40 years or older. NHIS responses regarding time since FIT-DNA test were not released in 2019 but were available for 2021. Because of this, CRC screening comparisons between years do not examine FIT-DNA use or overall CRC estimates (which include FIT-DNA).

**Table 1:** Definitions of Being Up to Date With Breast, Cervical, and Colorectal Cancer Screening

Screening type	Definition of up to date
Breast cancer	Mammogram within prior 2 years
Cervical cancer	Pap within prior 3 years (aged 21–65 y), or HPV test alone in prior 5 years (aged 30–65 y), or Pap + HPV co-test in prior 5 years (aged 30–65 y)
Colorectal cancer	Colonoscopy within 10 years, or flexible sigmoidoscopy within 5 years, or FOBT/FIT within 1 year, or CT colonography within 5 years, or FIT-DNA within 3 years

Abbreviations: CT, computed tomography; FIT-DNA, fecal immunochemical DNA test; FOBT, fecal occult blood test; HPV, human papillomavirus; Pap, Papanicolaou test.

Findings are national estimates of screening test use, reported as weighted percentages with 95% CIs. Survey design variables and survey weights were used in all analyses to account for the complex sample design and produce national estimates. We examined the percentage of US adults of screening age who were up to date with screenings for 2021 overall, and by sociodemographic and health care access characteristics, using Wald F-tests (significance set at *P* < .05). Overall estimates were also age-standardized to the projected 2000 US standard population (7). We compared age-standardized estimates of being up to date for 2021 and 2019. Estimates not meeting NCHS reliability standards were suppressed (8). We conducted analyses by using SAS version 9.4 (SAS Institute, Inc) and SUDAAN version 11.0.1 (RTI International).

## Results

In 2021, an estimated 75.7% (95% CI, 74.4%–76.9%) of US women were up to date with breast cancer screening, 75.2% (95% CI, 73.9%–76.4%) were up to date with cervical cancer screening, and 72.2% (95% CI, 71.2%–73.2%) of US adults were up to date with CRC screening (Tables 2 and 3). Colonoscopy was the most commonly used CRC screening test, with an estimated 63.1% (95% CI, 62.1%–64.2%) up to date, compared with 10.1% (95% CI, 9.4%–10.8%) for FOBT/FIT and 8.3% (95% CI, 7.7%–8.9%) for FIT-DNA (age-standardized, not mutually exclusive, not shown in tables). An estimated 15.5% (95% CI, 14.7%–16.3%) were up to date with any blood stool test (FOBT/FIT or FIT-DNA). Age-standardized breast and cervical screening estimates (Table 2) were below HP 2030 targets (80.5% and 84.3%, respectively) and below HP 2020 targets (81.1% and 93.0%, respectively). Assuming that all women aged 30 to 65 years with unknown HPV test use did or did not have an HPV test did not yield large differences in cervical cancer screening estimates (75.7% [95% CI, 74.4%–76.9%] vs 74.1% [95% CI, 72.8%–75.4%], data not shown). Age-standardized estimates of CRC screening test use (Table 3) exceeded the HP 2020 target (70.5%) and approached the HP 2030 target (74.4%).

**Table 2:** Percentage of Women Up to Date With Breast and Cervical Cancer Screening, United States, 2021

Characteristic	Breast cancer screening			Cervical cancer screening		
	No.	Weighted % (95% CI)	*P* value^a^	No.	Weighted % (95% CI)	*P* value^a^
**Overall, unadjusted**	6,327	75.7 (74.4–76.9)	NA	8,716	75.2 (73.9–76.4)	NA
**Overall, age-standardized^b^**	6,327	75.6 (74.4–76.8)	NA	8,716	75.5 (74.2–76.7)	NA
**Age, y**						
21–30	NA	NA	NA	1,759	67.8 (64.8–70.7)	<.001
31–40	NA	NA		2,308	82.3 (80.3–84.1)	
41–50	NA	NA		1,848	78.0 (75.5–80.3)	
51–65	NA	NA		2,801	73.5 (71.3–75.5)	
50–64	3,850	75.3 (73.8–76.8)	.44	NA	NA	NA
65–74	2,477	76.3 (74.3–78.2)		NA	NA	
**Race**						
AIAN	104	52.8 (42.5–63.0)	<.001	172	64.0 (51.3–75.4)	<.001
Asian	326	66.6 (60.2–72.5)		705	63.6 (59.1–68.0)	
Black/African American	766	81.6 (78.0–84.8)		1,106	73.3 (69.4–77.0)	
White	4,861	75.7 (74.3–77.1)		6,026	78.1 (76.6–79.6)	
Other, single and multiple race	55	73.4 (57.7–85.7)		166	72.5 (62.3–81.2)	
Missing/unknown	215	79.4 (72.7–85.1)		541	65.4 (60.4–70.1)	
**Ethnicity^c^**						
Non-Hispanic	5,694	75.9 (74.7–77.2)	.29	7,203	76.7 (75.3–78.1)	<.001
Hispanic	633	73.8 (69.8–77.5)		1,513	68.7 (65.6–71.6)	
Mexican/Mexican American	341	72.9 (67.3–78.1)		823	68.0 (64.4–71.4)	
Other Hispanic group^d^	282	74.3 (68.0–79.9)		673	69.7 (64.8–74.3)	
**Education**						
Less than high school	532	63.6 (58.3–68.7)	<.001	592	57.7 (52.6–62.8)	<.001
High school/GED	1,517	72.6 (70.0–75.1)		1,736	66.4 (63.4–69.3)	
Some college	1,916	76.5 (74.2–78.7)		2,356	74.2 (72.1–76.3)	
College degree	2,335	80.7 (78.8–82.4)		3,999	83.8 (82.4–85.1)	
Missing/unknown	27	—e		33	—e	
**Income, %^f^**						
≤138	1,060	64.8 (61.0–68.5)	<.001	1,558	67.4 (64.4–70.4)	<.001
>138–250	1,154	69.5 (65.9–72.9)		1,639	66.1 (63.1–69.0)	
>250–400	1,217	76.4 (73.4–79.3)		1,720	73.8 (70.9–76.6)	
>400	2,896	81.4 (79.7–83.0)		3,799	83.4 (81.8–84.9)	
**Duration of US residence, y**						
<10	53	61.3 (45.7–75.5)	.09	358	55.9 (49.3–62.3)	<.001
≥10	897	73.9 (70.4–77.2)		1,349	69.6 (66.7–72.4)	
Born in the US	5,260	76.5 (75.1–77.8)		6,797	77.7 (76.3–79.0)	
Missing/unknown	117	67.0 (55.2–77.4)		212	68.6 (60.3–76.1)	
**County urbanization level^g^**						
Large central metro	1,678	75.5 (72.9–77.9)	.02	2,903	73.1 (71.0–75.2)	.002
Large fringe metro	1,544	77.2 (74.7–79.6)		2,130	78.5 (76.0–80.7)	
Medium/small metro	2,097	76.3 (74.0–78.5)		2,568	76.1 (73.6–78.5)	
Nonmetropolitan	1,008	72.2 (69.3–74.9)		1,115	72.0 (68.2–75.6)	
**Sexual orientation**						
Lesbian or gay	97	78.8 (68.7–86.8)	.19	163	71.4 (62.8–78.9)	.03
Straight	5,976	76.0 (74.7–77.2)		7,867	76.0 (74.7–77.3)	
Bisexual	46	60.6 (45.1–74.6)		328	69.4 (62.4–75.9)	
Other	23	—e		73	59.6 (44.2–73.7)	
Missing/unknown	185	66.7 (57.3–75.2)		285	65.7 (58.7–72.3)	
**Usual source of care**						
Yes	5,774	78.6 (77.3–79.9)	<.001	7,222	78.3 (77.0–79.6)	<.001
No	547	44.1 (39.2–49.0)		1,490	61.1 (58.0–64.2)	
Missing/unknown	6	—e		4	—e	
**Insurance**						
Aged <65 y						
Private	2,807	80.1 (78.4–81.7)	<.001	5,999	79.8 (78.4–81.2)	<.001
Medicaid/other public	519	68.4 (63.4–73.1)		1,361	70.6 (67.3–73.8)	
Other coverage	233	76.8 (69.7–82.9)		341	70.1 (62.9–76.5)	
Uninsured	283	42.3 (35.3–49.6)		828	56.6 (52.4–60.8)	
Missing/unknown	8	—e		15	—e	
Aged ≥65 y^h^						
Private	968	78.9 (75.9–81.7)	<.001	85	70.1 (57.9–80.5)	.21
Medicare + Medicaid	191	74.7 (67.0–81.4)		10	—e	
Medicare Advantage	887	80.2 (76.9–83.2)		45	—e	
Medicare only	300	58.8 (51.6–65.7)		21	—e	
Other coverage	113	79.7 (70.4–87.2)		7	—e	
Uninsured	14	—e		4	—e	
Missing/unknown	4	—e		0	NA	
**Disability**						
Yes	782	65.8 (61.8–69.7)	<.001	513	64.0 (58.6–69.1)	<.001
No	5,545	77.0 (75.7–78.3)		8,203	75.8 (74.5–77.1)	
**Doing errands alone**						
At least some difficulty	591	65.0 (60.2–69.6)	<.001	492	60.5 (54.3–66.4)	<.001
No difficulty	5,735	76.8 (75.5–78.0)		8,224	76.1 (74.8–77.4)	
Missing/unknown	1	—e		0	NA	
**Wellness check within 3 years**						
Yes	6,066	78.1 (76.9–79.3)	<.001	8,180	77.8 (76.5–79.0)	<.001
No	249	13.8 (9.6–19.0)		511	36.5 (31.6–41.5)	
Missing/unknown	12	—e		25	—e	

Abbreviations: AIAN, American Indian or Alaska Native and includes AIAN only or in combination with another race; GED, general educational development; NA, not applicable; NCHS, National Center for Health Statistics; NHIS, National Health Interview Survey; USPSTF, US Preventive Services Task Force.

a *P* value from Wald F tests, testing for any differences across groups excluding missing or unknown.

b Overall percentages are presented unadjusted and age-standardized to the 2000 US standard population. Percentages were age-standardized using the following age groups: 50–64, 65–74 (breast); and 21–34, 35–44, 45–65 (cervical). Percentages by sociodemographic and other variables are unadjusted.

c *P* value reflects differences between Hispanic and non-Hispanic groups.

d Information about adults from other Hispanic origin or ethnicity groups is not available in the NHIS public-use file.

e Estimates suppressed because they did not meet NCHS reliability standards (8).

f Family income as a percentage of the federal poverty threshold, and multiply imputed by NCHS when missing (5,9).

g Includes 4 groups based on the 2013 NCHS Urban–Rural Classification Scheme for Counties (5,10).

h For cervical screening, includes only age 65 years because USPSTF does not recommend routine screening beyond this age.

**Table 3:** Percentage of Adults Up to Date With Colorectal Cancer Screening, United States, 2021

Characteristic	Colorectal cancer screening		
	No.	Weighted % (95% CI)	*P* value^a^
**Overall (unadjusted)**	12,520	72.2 (71.2–73.2)	NA
**Overall (age-standardized)^b^**	12,520	71.6 (70.6–72.6)	NA
**Age, y**			
50–64	7,338	66.1 (64.8–67.4)	<.001
65–75	5,182	83.0 (81.7–84.3)	
**Sex**			
Male	5,689	71.1 (69.6–72.5)	.03
Female	6,830	73.3 (72.0–74.6)	
Unknown	1	—c	
**Race**			
AIAN	208	62.6 (55.0–69.8)	<.001
Asian	611	60.9 (55.7–65.8)	
Black/African American	1,399	71.3 (68.2–74.2)	
White	9,776	74.0 (72.9–75.1)	
Other single and multiple race	109	64.2 (52.9–74.5)	
Missing/unknown	417	63.0 (57.0–68.6)	
**Ethnicity^d^**			
Non-Hispanic	11,304	73.6 (72.6–74.6)	<.001
Hispanic	1,216	62.1 (58.7–65.4)	
Mexican/Mexican American	662	58.8 (54.2–63.3)	
Other Hispanic group^e^	536	66.0 (61.1–70.6)	
**Education**			
Less than high school	1,071	59.2 (55.7–62.7)	<.001
High school/GED	3,120	67.5 (65.6–69.5)	
Some college	3,611	73.5 (71.7–75.3)	
College degree	4,663	78.4 (77.0–79.8)	
Missing/unknown	55	76.2 (60.6–87.9)	
**Income, %^f^**			
≤138	1,913	60.3 (57.5–63.1)	<.001
>138–250	2,176	65.2 (62.7–67.8)	
>250–400	2,420	71.3 (68.9–73.5)	
>400	6,012	78.6 (77.4–79.8)	
**Duration of US residence**			
<10 y	83	37.6 (26.2–50.1)	<.001
≥10 y	1,705	64.9 (62.0–67.7)	
Born in US	10,467	74.5 (73.5–75.5)	
Missing/unknown	265	60.1 (52.2–67.7)	
**County urbanization level^g^**			
Large central metro	3,355	71.7 (69.7–73.6)	.02
Large fringe metro	3,027	74.2 (72.4–76.0)	
Medium and small metro	4,121	72.6 (70.6–74.5)	
Nonmetropolitan	2,017	69.3 (66.7–71.9)	
**Sexual orientation**			
Lesbian or gay	250	76.1 (69.1–82.3)	.65
Straight	11,744	72.4 (71.4–73.5)	
Bisexual	90	70.0 (58.4–80.0)	
Other	41	—c	
Missing/unknown	395	65.1 (58.7–71.1)	
**Usual source of care**			
Yes	11,240	75.5 (74.5–76.5)	<.001
No	1,274	42.9 (39.7–46.1)	
Missing/Unknown	6	—c	
**Insurance**			
Age <65 y			
Private	5,265	71.0 (69.5–72.4)	<.001
Medicaid/other public	911	57.4 (53.8–61.0)	
Other coverage	535	75.2 (70.6–79.4)	
Uninsured	607	29.8 (25.3–34.6)	
Missing/unknown	20	—c	
Age ≥65 y			
Private	2,067	86.3 (84.4–88.0)	<.001
Medicare + Medicaid	337	71.1 (63.7–77.7)	
Medicare Advantage	1,720	85.4 (83.4–87.3)	
Medicare only	619	74.1 (69.8–78.0)	
Other coverage	399	84.8 (80.4–88.6)	
Uninsured	30	—c	
Missing/unknown	10	—c	
**Disability**			
Yes	1,452	71.6 (68.8–74.3)	.63
No	11,068	72.3 (71.3–73.4)	
**Doing errands alone**			
At least some difficulty	1,015	68.5 (65.1–71.8)	.02
No difficulty	11,502	72.6 (71.5–73.6)	
Missing/unknown	3	—c	
**Wellness check within 3 years**			
Yes	11,884	75.1 (74.1–76.1)	<.001
No	603	17.3 (14.0–20.9)	
Missing/unknown	33	—c	

Abbreviations: AIAN, American Indian/Alaska Native and includes AIAN only or in combination; GED, general educational development; NA, not applicable; NCHS, National Center for Health Statistics; NHIS, National Health Interview Survey; USPSTF, US Preventive Services Task Force.

a *P* value from Wald F tests testing for any differences across groups excluding missing/unknown.

b Overall percentages are presented as unadjusted and age-standardized to the 2000 US standard population. Percentages were age-standardized using the following age groups: 50–64, 65–75. Percentages by sociodemographic and other variables are unadjusted.

c Estimates suppressed because they did not meet NCHS standards for reliability (8).

d *P* value reflects differences between Hispanic and non-Hispanic groups.

e Information about adults from other Hispanic origin or ethnicity groups is not available in the NHIS public-use file.

f Family income as a percentage of the federal poverty threshold, and multiply imputed by NCHS when missing (5,9).

g Includes 4 groups based on the 2013 NCHS Urban–Rural Classification Scheme for Counties (5,10).

Estimates were below 50% among those without a wellness check in 3 years across all screening types, among those without a usual source of care or insurance (aged <65 years) for breast and CRC screening, and among those residing in the US for less than 10 years for CRC screening. American Indian or Alaska Native (AIAN) and Asian adults tended to have lower estimates by race across screening types. Hispanic adults were less likely than non-Hispanic adults to be up to date with cervical and CRC screening. Significant differences existed across all screening types by urbanization level, education, and income (Tables 2 and 3).

Age-standardized estimates of being up to date with breast and cervical cancer screening were similar in 2021 and 2019 (75.6% [95% CI, 74.4%–76.8%] vs 76.2% [95% CI, 74.9%–77.5%], *P* = .51 for breast cancer screening and 75.5% [95% CI, 74.2%–76.7%] vs 76.8% [95% CI, 75.6%–77.9%], *P* = .09 for cervical cancer screening). Colonoscopy estimates were also similar (63.1% [95% CI, 62.1%–64.2%] in 2021 vs 62.4% [95% CI, 61.3%–63.4%] in 2019, *P* = .28). Estimated FOBT/FIT use was somewhat higher in 2021 (10.1% [95% CI, 9.4%–10.8%] in 2021 vs 6.6% [95% CI, 6.1%–7.1%] in 2019, *P* < .001).

## Discussion

Estimates suggest that approximately one-quarter of US adults of screening age were not up to date with breast, cervical, and CRC screening in 2021, and screening test use was below HP 2030 targets (4); CRC test use neared the target and exceeded the HP 2020 target (11). Colonoscopy was the most common CRC test, although 15% of adults were estimated to have received a stool blood test. Adults who were uninsured, without a usual source of care, or with shorter residence in the US had low screening uptake, consistent with previous evidence (2,12,13). By race, estimates for Asian and AIAN adults tended to be lowest across screening types. Hispanic adults were less likely to be up to date than non-Hispanic adults for cervical and CRC screening. Differences were also observed by education, income, and urbanization level.

The similarity in most up to date test estimates for 2021 and 2019 could reflect recommended screening intervals longer than 1 year, as well as recovery from reported declines in screening use during the COVID-19 pandemic (14–19). Higher FOBT/FIT estimates for 2021 may suggest a shift toward increased home stool testing for CRC screening during the pandemic, consistent with other studies (20,21). Home-based testing has been identified as a screening facilitator during the pandemic (22). However, differences in survey methods and questions for nonendoscopic screening tests could have affected estimates.

Findings are subject to limitations. Data were self-reported, which could result in reporting bias (eg, recall bias). Limited evidence exists about self-reported HPV test accuracy (23,24). Assuming that all women aged 30 to 65 years with unknown HPV test use either did or did not have an HPV test did not yield large differences in cervical cancer screening estimates. Weights were adjusted for nonresponse, although nonresponse bias may be possible. Consistent with previous studies and HP targets (2–4,12,13,25), tests performed for any reason were included. Caution may be warranted in interpreting findings for small subgroups given missing information for some variables.

Approximately 1 in 4 adults of screening age were not up to date with breast, cervical, and CRC screening recommendations in 2021. Estimates were below current national targets and disparities persisted across sociodemographic and health care access groups, with particularly low use among those with less health care access. Use of these tests within recommended screening intervals may approximate prepandemic levels. FOBT/FIT use may have increased modestly. Survey changes could have affected estimates although findings are consistent with previous evidence (20,21).
